# Adverse Side Effects of Crizotinib in the Treatment of Anaplastic Lymphoma Kinase-Mutated Non-small Cell Lung Cancer: A Systematic Review

**DOI:** 10.7759/cureus.45517

**Published:** 2023-09-19

**Authors:** Sherie George, Srushti R Shahi, Zahra Ali, Abdelrahman Abaza, Aneeque Jamil, Sai Dheeraj Gutlapalli, Marya Ali, Mrinal J P Oble, Shamsun Nahar Sonia, Pousette Hamid

**Affiliations:** 1 General Medicine, California Institute of Behavioral Neurosciences & Psychology, Fairfield, USA; 2 Medicine, California Institute of Behavioral Neurosciences & Psychology, Fairfield, USA; 3 Pathology, California Institute of Behavioral Neurosciences & Psychology, Fairfield, USA; 4 Internal Medicine, California Institute of Behavioral Neurosciences & Psychology, Fairfield, USA; 5 Psychiatry, California Institute of Behavioral Neurosciences & Psychology, Fairfield, USA; 6 Neurology, California Institute of Behavioral Neurosciences & Psychology, Fairfield, USA

**Keywords:** alk positive, adverse side effect, crizotinib, alk inhibitor, advanced non-small cell lung cancer

## Abstract

Lung cancer is the leading cause of cancer deaths worldwide, with the majority consisting of non-small cell lung cancer (NSCLC). Genetic mutations present an opportunity for targeted therapy, in addition to current mainstay treatments such as chemotherapy and radiotherapy. Overall, 5% of NSCLCs have an anaplastic lymphoma kinase (ALK) mutation, often prevalent in a younger population. Crizotinib is an ALK inhibitor that was approved to treat ALK-mutated advanced NSCLC. While common side effects such as nausea, fatigue, and diarrhea are mostly well tolerated, adverse side effects can lead to treatment discontinuation or adjustment or can be fatal. This systematic review used articles searched on Google Scholar and PubMed which were assessed using the Cochrane risk-of-bias tool and Newcastle-Ottawa Scale. This yielded nine papers consisting of randomized controlled trials and cohort studies. Side effects resulting in cessation of treatment or dose reduction included liver dysfunction, nausea, neutropenia, and QT prolongation. This review showed that crizotinib has a better side effect profile than chemotherapy in ALK-positive NSCLC, even though toxicities leading to treatment withdrawal are present. Adverse effects were tackled by dose reduction, temporary withdrawal from treatment, and close monitoring.

## Introduction and background

In 2020, lung cancer was the primary cause of cancer deaths in both men and women, resulting in 18% of all cancer deaths. Globally, there were 2,200,000 newly diagnosed lung cancer cases and 1,800,000 deaths from lung cancer alone. It remains one of the most diagnosed types of cancer worldwide, causing one in five deaths [1]. Non-small cell lung cancer (NSCLC) forms the majority of lung cancers, accounting for 80-85% of all diagnosed lung cancers. There are three main types of NSCLC, namely, squamous cell, large cell, and adenocarcinomas, which account for 40% of all NSCLCs [2]. 

Research over the years has identified genetic mutations present in NSCLCs that have allowed targeted systemic therapy. Overall, 25-30% of all lung adenocarcinomas have Kirsten rat sarcoma viral oncogene homolog (KRAS) mutations, 15-20% have epidermal growth factor (EGFR) mutations, and 5% have anaplastic lymphoma kinase (ALK) mutations, while other mutations such as ROS1, MET1, and BRAF are present at a lower incidence [3]. Clinical trials have worked toward developing drugs that target these oncogenic driver pathways, providing an alternative form of treatment to chemotherapy, to create molecular targeted therapies.

ALK mutations were seen to be more prevalent in younger patients, and those who had never smoked or were light smokers. They are not only prevalent in NSCLCs but also in neuroblastomas and large B-cell lymphomas, suggesting a role in tumor progression [4]. The gold standard to identify ALK mutations is fluorescence in situ hybridization, which was the first method to be approved by the Food and Drug Administration (FDA). Additionally, next-generation sequencing is useful to identify ALK fusion mutations such as EML4-ALK and co-existing mutations that would be of clinical relevance while planning treatment, allowing more patients to be eligible for ALK-targeted therapy [5,6].

Crizotinib is a tyrosine kinase inhibitor (TKI) that was approved by the FDA in 2011 to treat ALK-mutated locally advanced or metastatic NSCLC after clinical trials showed good objective response rates and increased progression-free survival [4]. Also known by its trade name Xalkori, crizotinib works as an ATP-competitive inhibitor of ALK, ROS1, and MET tyrosine kinase receptors. It is usually prescribed at 250 mg twice a day [7]. Two initial phase I clinical trials leading to the approval of crizotinib prescribed the drug at 250 mg twice a day to 255 ALK-positive patients, who had metastatic or locally advanced NSCLC. The average age across both studies was 51.5 years, with the majority of patients being white and non-smokers. The objective response rate across both studies was 55%, which was mostly reached within the first eight weeks of commencing treatment. The average response period was 42 and 48 weeks in each of the studies [7,8]. The efficacy of crizotinib was established in a phase III trial, where 347 patients were assigned to either chemotherapy (pemetrexed or docetaxel) or crizotinib treatment. Patients on chemotherapy with advanced NSCLC were permitted to receive crizotinib treatment if they wished to cross over. Patients who received crizotinib had a greater progression-free survival of 7.7 months compared to three months in patients who received chemotherapy. Overall, 64% of patients who received chemotherapy also crossed over to crizotinib treatment [8].

Common side effects of crizotinib include nausea, diarrhea, vomiting, visual changes, fatigue, infection, loss of appetite, and several others. While mostly well tolerated, crizotinib can also cause adverse side effects (Grades 3-5) that lead to the discontinuation of treatment and poor quality of life [9]. This should be taken into consideration when deciding treatment for those who may benefit from another drug with a better side effect profile.

This systematic review aims to discuss the adverse side effect profile (Grade 3 and above) of crizotinib by analyzing several studies from across the world. It aims to discuss the side effects leading to dose changes or discontinuation of treatment, as well as how this can be addressed.

## Review

Methodology

This systematic review was performed following the Preferred Reporting Items for Systematic Reviews and Meta-Analyses 2020 checklist guidelines [10].

Databases

Google Scholar and PubMed were searched to collect relevant papers for inclusion in the systematic review.

Inclusion Criteria

Each article was assessed by two independent investigators (SG and ZA) to determine its relevance by screening the article title and abstract. The following inclusion criteria were used to screen articles found: (1) studies published in the English language; (2) human studies; (3) randomized controlled trials and cohort studies; (4) articles published in the last 10 years; (5) articles including males and females above 18 years of age, worldwide; and (6) randomized clinical trials and cohort studies that assessed the effects of crizotinib in ALK-mutated NSCLC.

Exclusion Criteria

Studies that had the following criteria were excluded: (1) systematic reviews, pharmaceutical reports, cross-sectional studies, meta-analyses, case reports, case series, and letters to the editor; (2) articles not in the English language; (3) articles that were not peer-reviewed; (4) studies including pediatric patients; (5) gray literature; (6) studies that did not assess ALK-mutated NSCLC.

Data Extraction

Two investigators (SG and ZA) worked independently to extract data from selected studies. Study design, number of participants, side effects from crizotinib, and study outcomes were recorded for each study.

Quality Assessment Tools

Two investigators (SG and ZA) evaluated the risk of bias for each study. Cochrane risk-of-bias tool was used to assess randomized clinical trials (Table 1) and the Newcastle-Ottawa Scale was used to assess cohort studies (Table 2). Only studies that had a low risk of bias and moderate or high quality were included in this systematic review. Any disagreement was discussed between the first and second authors. In case of disputes, a third independent reviewer made the decision.

**Table 1:** Quality assessment of randomized controlled trials using the Cochrane risk-of-bias tool.

RCT	Random sequence generation	Allocation concealment	Blinding	Incomplete outcome data assessed	Uniform and explicit outcome reporting	Free of selective outcome reporting	Overall risk of bias
Solomon et al. 2014 [11]	Unsure	Yes	No	Yes	Yes	Yes	Low
Peters et al. 2017 [12]	Yes	No	No	Yes	Yes	Yes	Low
Camidge et al. 2018 [13]	Unclear	Yes	No	Yes	Yes	Yes	Low

**Table 2:** Quality assessment of cohort studies using the Newcastle-Ottawa Scale.

Study	Selection	Comparability	Outcome	Overall
Del Valle and Chang 2019 [14]	**	**	**	6, moderate
Mohieldin et al. 2018 [15]	**	**		4, moderate quality
Kapoor et al. 2022 [16]	***	**	**	7, high quality
Liu et al. 2019 [17]	***	**	**	7, high quality
Rosa et al. 2022 [18]	**	**	**	6, moderate quality
Ueno et al. 2019 [19]	**	**	**	6, moderate quality

Results

Literature Search and Study Selection

In Google Scholar, searching “Adverse Effects of Crizotinib in Patients With ALK-Positive Advanced NSCLC” yielded 11,400 results. Applying filters for the last 10 years (2012-2022) and additional filters “Crizotinib” and “ALK” the title produced 1,100 papers. The first 76 papers were considered to apply the inclusion and exclusion criteria, as papers past this number were irrelevant for the review.

**Figure 1:**
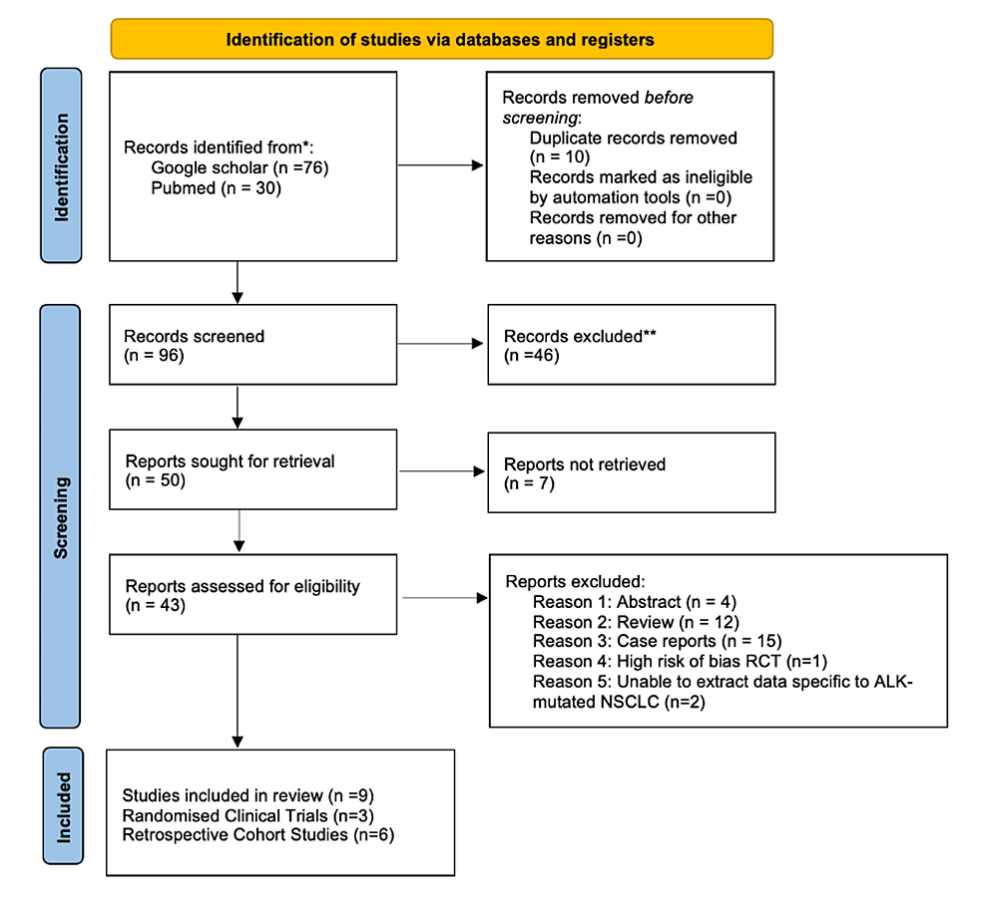
Preferred Reporting Items for Systematic Reviews and Meta-Analyses flow diagram.

The Snowball technique was then used to retrieve 30 additional papers. In PubMed, “Adverse Effects of Crizotinib in Patients With ALK-Positive Advanced NSCLC” was searched and the technique was applied to highly relevant articles to find other articles. Papers were obtained from November 27, 2022, to November 30, 2022. Duplicates of articles found from Google Scholar and PubMed were removed manually on Excel. The MeSH technique was tried but did not yield relevant papers on PubMed. This yielded a total of 106 papers (Figure 1). Only full-text articles and articles that were accessible for free were included.

After duplicates were removed and inclusion and exclusion criteria were applied, nine papers including three randomized clinical trial articles and six cohort studies were selected. Randomized clinical trials were analyzed with the Cochrane quality assessment tool and cohort studies were assessed using the Newcastle Ottowa Scale to assess the level of bias, as shown in Table 1 and Table 2, respectively. Grade 3 to 5 adverse effects from crizotinib treatment in each study were extracted, as shown in Table 3 and Table 4.

**Table 3:** Baseline characteristics and Grades 3-5 adverse effects from randomized controlled trials. ALT: alanine aminotransferase; AST: aspartate aminotransferase; GGT: Gamma-glutamyltransferase; CK: creatine kinase

Study	Study design	Number of patients in the crizotinib group	Number of patients in the corresponding group (different treatment or no treatment)	Percentage of patients affected by adverse effects of corresponding treatment (Grades 3–5)		Percentage of patients affected by adverse effects of crizotinib (Grades 3–5)	
Solomon et al. 2014 [11]	Randomized clinical trial	171	Chemotherapy: 169	Vision disorders	0	Vision disorders	1
				Diarrhea	1	Diarrhea	2
				Edema	1	Edema	1
				Vomiting	3	Vomiting	2
				Constipation	0	Constipation	2
				Elevated ALT	2	Elevated ALT	14
				Headache	1	Headache	1
				Fatigue	2	Fatigue	3
				Neutropenia	15	Neutropenia	11
				Stomatitis	1	Stomatitis	1
				Leukopenia	5	Leukopenia	2
				Nausea	2	Nausea	1
				Decreased appetite	1	Decreased appetite	2
				Neuropathy	0	Neuropathy	1
				Dyspnea	2	Dyspnea	3
				Thrombocytopenia	7	Thrombocytopenia	0
				Upper respiratory infection	0	Upper respiratory infection	1
				Pyrexia	1	Pyrexia	0
				Dizziness	1	Dizziness	0
				Anemia	9	Anemia	0
Peters et al. 2017 [12]	Randomized clinical trial	151	Alectinib: 152	Nausea	1	Nausea	3
				Diarrhea	0	Diarrhea	2
				Vomiting	0	Vomiting	3
				ALT increased	5	ALT increased	15
				AST increased	5	AST increased	11
				GGT increased	1	GGT increased	1
				Edema	0	Edema	1
				Anemia	5	Anemia	1
Camidge et al. 2018 [13]	Randomized clinical trial	138	Brigatinib: 137	Diarrhea	1	Diarrhea	2
				Increase CK level	16	Increase CK level	1
				Nausea	1	Nausea	3
				Hypertension	10	Hypertension	3
				Increased ALT	1	Increased ALT	9
				Increased lipase level	13	Increased lipase level	5
				Vomiting	1	Vomiting	2
				Constipation	0	Constipation	1
				Increased amylase level	5	Increased amylase level	1
				Pruritis	1	Pruritis	1
				Decreased appetite	1	Decreased appetite	3
				Peripheral edema	1	Peripheral edema	1
				Upper abdominal pain	1	Upper abdominal pain	1
				Bradycardia	1	Bradycardia	0
				Pain in the extremity	0	Pain in the extremity	1
				Increase in creatinine levels	0	Increase in creatinine levels	1
				Neutropenia	0	Neutropenia	4
				Pleural effusion	1	Pleural effusion	1
				Photopsia	0	Photopsia	1

**Table 4:** Baseline characteristics and adverse effects from cohort studies.

Study	Study design	Median age of the crizotinib group	Number of patients in the crizotinib group	Number of patients in the corresponding group (different treatment or no treatment)	Duration of treatment	Adverse effects of crizotinib (Grades 3–5)	
Del Valle and Chang 2019 [14]	Retrospective cohort study	42	22	NA		Bradycardia	4.5%
						Prolonged QTc	4.5%
						Complete heart block	4.5%
						Nausea, vomiting	4.5%
						Transaminitis	4.5%
						Pneumonitis	4.5%
						Neutropenia	13.6%
Mohieldin et al. 2018 [15]	Retrospective cohort study	53	38	NA	4 years 8 months	Fatigue	2.6%
						Peripheral edema	5.3%
						Transaminitis	7.9%
						Diarrhea	7.9%
						Prolonged QT	7.9%
Kapoor et al. 2022 [16]	Retrospective cohort study	50	188	NA	49.4 months	Transaminitis	4.8%
						QTc prolongation	4.2%
Liu et al. 2019 [17]	Retrospective cohort study	49.5	104	NA	4 years	Elevated transaminases	4.8%
						Neutropenia	2.9%
						Anemia	1%
Rosa et al. 2022 [18]	Observational prospective and retrospective cohort study	60.5	91	NA	2years 7 months	Elevated transaminase levels	4.4%
						Infections and infestations	2.2%
						Benign neoplasia	1.1%
						Bradycardia	1.1%
						Skin disorders	1.1%
						Leukopenia	1.1%
						Neutropenia	4.4%
						Nervous system disorders	1.1%
						Gastrointestinal disorders	4.4%
						Visual disorders	1.1%
						Renal and urinary disorders	1.1%
						Respiratory disorders	2.2%
Ueno et al. 2019 [19]	Retrospective cohort study	61	2028	NA	52 weeks	Gastrointestinal disorders	8.8%
						Eye disorders	0.6%
						Hepatobiliary disorders	10.2%
						Nervous system disorders	1.3%
						Metabolism and nutrition disorders	4.7%
						Respiratory, thoracic and mediastinal disorders	4.6%
						Infections and infestations	4.6%
						Skin and subcutaneous tissue disorders	0.4%
						Blood and lymphatic system disorders	3.9%
						Cardiac disorders	1%
						Renal and urinary disorders	0.7%
						Psychiatric disorders	0.5%
						Vascular disorders	0.9%
						Musculoskeletal and connective tissue disorders	0.2%
						Neoplasms benign, malignant, and unspecified (including cysts and polyps)	1%
						Endocrine disorders	0.1%
						Congenital, familial, and hereditary disorders	0.1%

Further data on the percentage of patients in each study who required dose reduction, for whom treatment was temporarily withheld, or who withdrew from crizotinib therapy is summarized in Table 5.

**Table 5:** Percentage of patients who required dose adjustments or withdrew from crizotinib therapy.

Study	Number of patients on crizotinib therapy	Percentage of patients who had to undergo dose adjustments due to adverse effects (temporarily withheld, reduced)	Percentage of patients who permanently withdrew from crizotinib therapy due to adverse effects
Solomon et al. 2014 [11]	171	14	12
Peters et al. 2017 [12]	151	46	13
Camidge et al. 2018 [13]	138	21	9
Del Valle and Chang 2019 [14]	22	27.3	13.6
Mohieldin et al. 2018 [15]	38	15.8	-
Kapoor et al. 2022 [16]	188	-	2.7
Liu et al. 2019 [17]	104	1.92	0.96
Rosa et al. 2022 [18]	91	42.8	11.4
Ueno et al. 2019 [19]	2028	38.4	26

Table 6 and Table 7 demonstrate the percentage of patients who had to withdraw from treatment or required dose changes during treatment, secondary to adverse effects of crizotinib, in the Japan cohort study [19] and Camidge et al. (2018) study [13], respectively.

**Table 6:** Adverse effects from the Japan cohort study resulting in dose changes or withdrawal from treatment.

Adverse effect (>Grade 3)	Number of patients	Patients who required dose reduction (%)	Patients who withdrew from treatment (%)
Hepatobiliary problems	206	62.6	31.6
Gastrointestinal problems	178	48.9	24.7
Respiratory, thoracic, and mediastinal problems	121	12.4	76.9
Metabolism and nutrition problems	96	41.7	29.2
Infections	94	44.7	38.3
Blood and lymphatic problems	78	52.6	10.3
Nervous system problems	27	40.7	37
Neoplasms	21	4.8	71.4
Cardiac problems	21	42.9	42.9

**Table 7:** Percentage of patients who required dose reduction due to adverse side effects. From Camidge et al.’s randomized controlled trial [13].

Adverse side effects of all grades	Percentage of patients who required dose reduction (%)
Increased blood creatine phosphokinase	1.5
Increased alanine aminotransferase	5.8
Increased aspartate aminotransferase	0.7
Increased lipase level	0.7
Nausea	4.4
Diarrhea	2.2
Decreased appetite	1.5
Vomiting	1.5
Peripheral edema	2.2
Pneumonitis	0.7

Discussion

Many adverse effects from crizotinib treatment have been highlighted in the studies above, particularly toxicities above Grade 3. The Common Terminology Criteria for Adverse Events was used to categorize any adverse side effects, and the European Organisation for Research and Treatment of Cancer (EORTC) core quality-of-life questionnaire (QLQ-C30) was used to assess the overall quality of life.

In the Solomon et al. study [11], 5% of patients treated with crizotinib had to cease treatment due to adverse side effects compared to 8% in the chemotherapy group. This is inclusive of four patients who withdrew from crizotinib treatment due to an increase in alanine aminotransferase (ALT) levels, with one patient suffering a Grade 2 liver injury. In other patients, to combat an increase in ALT levels, the dose of crizotinib was decreased or interrupted. One incidence of deadly pneumonitis ended in death, although it should be emphasized that the patient was initially treated with chemotherapy before crizotinib. Additionally, two patients on crizotinib experienced interstitial lung disease, leading to cessation of treatment. However, patients on crizotinib had a progression-free survival of 10.9 months compared to seven months in the chemotherapy group. As seen in Table 3, while crizotinib had a higher number of patients suffering from an increase in ALT levels, the chemotherapy group had a higher rate of neutropenia, thrombocytopenia, and leukopenia. Furthermore, patients receiving crizotinib treatment had a higher quality of life than those receiving chemotherapy or no treatment at all. Looking at symptom management, crizotinib was superior in combating pain, shortness of breath, cough, and insomnia symptoms. With fewer side effects and the capacity to preserve the quality of life while boosting progression-free survival and response rates, crizotinib was a superior first-line treatment to chemotherapy for ALK-mutant NSCLC [11].

Peters et al. [12] conducted a phase three clinical trial to compare alectinib with crizotinib in treating previously untreated metastatic ALK-mutated NSCLC. Overall, 41% of alectinib patients compared to 50% of crizotinib patients experienced Grade 3-5 adverse effects. Most significantly, 15% and 11% of crizotinib patients had a Grade 3-5 rise in ALT and aspartate aminotransferase (AST), respectively, compared to 5% in both markers in the alectinib group. Other Grade 3-5 side effects in the crizotinib group occurred at a lower rate (1-3%) such as gamma-glutamyltransferase increase, peripheral edema, diarrhea, nausea, vomiting, and anemia. The trial reported two deaths with crizotinib compared to no deaths with alectinib, secondary to treatment. Overall, a higher proportion of crizotinib patients needed dose reduction and discontinued treatment permanently or temporarily compared to alectinib due to adverse side effects. Overall, alectinib had a lower toxicity and safer side effects profile compared to crizotinib, even though treatment with alectinib was longer at 17.9 months compared to 10.7 months.

A similar phase 3 trial by Camidge et al. [13] compared the efficacy and toxicity of brigatinib and crizotinib. Brigatinib is an ALK and ROS1 inhibitor, prescribed at 180 mg once daily. Overall, 61% of patients treated with brigatinib had a Grade 3 or higher adverse effect compared to 55% in the crizotinib group. Overall, 9% of crizotinib patients had an increase in ALT levels of Grade 3 or higher compared to 1% in the brigatinib group. Other side effects, including diarrhea, elevated creatinine kinase levels, nausea, hypertension, elevated lipase levels, vomiting, constipation, elevated amylase levels, pruritis, decreased appetite, peripheral edema, upper abdominal pain, pain, elevated blood creatinine levels, neutropenia, pleural effusion, and photopsia, all occurred in fewer than 5% of crizotinib patients. Moreover, 9% and 12% of patients on crizotinib and brigatinib, respectively, withdrew from treatment as a result of adverse side effects.

A small cohort study of 22 ALK-positive patients in Singapore [14] reported Grade 3 or higher toxicities including nausea and vomiting, bradycardia, prolonged QTc, complete heart block, transaminitis, and pneumonitis in one patient in each category. Additionally, three patients had Grade 3 or higher neutropenia, which led to treatment being withheld in one patient. Overall, three patients withdrew from treatment after a trial of dose reduction as a result of being unable to cope with vomiting, abdominal pain, and pneumonitis. This study shed light on the cardiotoxic effects (bradycardia, QTC prolongation, complete heart block) of crizotinib that occurred in four patients (18%). By withholding treatment, QTc prolongation and bradycardia effects were reversed. Two individuals experienced Grades 1 and 2 bradycardia. However, the patient who had a complete heart block needed a pacemaker to be inserted, after which he was able to restart treatment.

Similar results were seen in the studies by Solomon et al. [11] and Shaw et al. [20], where 2-4% of patients had Grade 3 or 4 QTc prolongation and 1% had a Grade 5 arrhythmia. To tackle bradycardia, Ou et al. 2013 [21] suggested that crizotinib should be withheld if the patient is symptomatic until the heart rate is at least 60 beats per minute or Grade 1. Interestingly, the study also suggested that bradycardia is associated with a greater response to treatment.

In total, 38 patients from 2013 to 2017 across two hospitals in Saudi Arabia and Kuwait were treated with crizotinib 250 mg twice a day [15]. While 84.2% of patients were able to continue with the 250 mg twice daily dose throughout the study, 15.8% of patients had to have a change in dose or temporarily withheld due to treatment toxicity. Common toxicities were fatigue, peripheral edema, and transaminitis. Grade 3 transaminitis affected two patients, and Grade 4 transaminitis affected one. After the enzyme levels returned to normal, therapy was restarted at a lower dose of 200 mg twice a day. Three patients had bradycardia above Grade 3, with prolonged QT interval, one of whom withdrew from treatment due to critical bradycardia that resolved three days later. The patient restarted treatment after her pacemaker was adjusted. The remaining two patients were switched to a lower dose of crizotinib.

When discussing therapy choices for advanced NSCLC, Kapoor et al. [16] discussed how patients in low-to-middle-income countries may not always have easy access to ALK inhibitors. The retrospective study that was conducted in Mumbai, India included 188 patients from March 2014 to December 2016. In terms of toxicities, 4.8% of patients had Grade 3 or 4 transaminitis, 4.2% had Grade 3 QTc prolongation, and 4.8% had renal cyst formation. Further, 2.7% of patients ceased treatment secondary to toxicity, while 1.1% of patients withdrew from treatment due to financial difficulties in affording treatment.

Crizotinib was approved in China to treat NSCLC in ALK-positive patients in 2013. Medical records at the Fudan University Shanghai Cancer Center from 2014 to 2018 of patients who had ALK-positive NSCLC and who had received crizotinib treatment were reviewed. Common side effects included increased transaminase levels, with 4.8% being at least Grade 3 in severity. This resulted in one patient discontinuing treatment due to Grade 4 effects, and two patients who experienced Grade 3 effects changing doses from 250 mg to 200 mg twice a day. Moreover, 2.9% of patients had Grade 3 neutropenia and 1% had anemia of Grade 3 or more. Other common side effects of lower grades included an increase in creatinine levels at 26.9% and diarrhea at 19.2% [17].

A study from Spain was the first in the country to examine the effectiveness and safety of crizotinib treatment in ALK-positive patients with advanced NSCLC [18]. The study was conducted from 2016 to 2019, including patients across 23 hospitals in Spain and a total of 91 patients. Overall, 57.1% of patients were able to tolerate crizotinib at the usual dosing of 250 mg bi-daily and did not need the dose to be withheld or decreased. However, in 22.4% of patients, the dose needed to be decreased, and in 18.6%, treatment needed to be withheld for an average of 12 days before being restarted. Moreover, 62.5% of patients experienced treatment-related side effects, 19.8% of which were Grade 3 to 4.

In terms of Grade 3 or 4 toxicities, 3.3% had elevated transaminase levels, 2.2% had an infection, 4.4% had neutropenia and gastrointestinal problems, 2.2% had respiratory problems, and 1.1% had renal/urinary problems, visual issues, nervous system issues, leukopenia, skin disorders, bradycardia, and benign neoplasms. One patient had a Grade 5 increase in transaminase level. Treatment was ceased in one patient after two months of treatment, who later died due to liver failure secondary to increased transaminase levels. Patients scored an average of 50 on the global quality of life scale before treatment, which increased to 62.5 after the first cycle of crizotinib. Shortness of breath and peripheral neuropathy were the two symptoms that were noted to worsen quality of life during treatment. While 57.1% of patients reported that crizotinib was well tolerated, 11.4% discontinued treatment because of adverse effects, similar to the study by Solomon et al. [11].

Ueno et al. [19] followed 2,028 ALK-positive NSCLC patients from 2012 to 2014 in Japan, who were treated with crizotinib. Overall, 91.6% of patients experienced side effects, most of which were Grade 1 or 2. At six months and 12 months of crizotinib treatment, 55.2% and 36.1% of patients remained on treatment. Factors that were highlighted to contribute significantly to early withdrawal from treatment included if patients were elderly, weighed less than 40 kg, had a body surface area of less than 1.2 m^2^, patients with a performance status (ECOG score) of 2 to 4, those who were previously exposed to asbestos or pneumoconiosis through their occupation of environment, and those who had a high Brinkman index, indicating a high smoking history. While 94.2% of patients were treated with 500 mg once a day, the dose had to be decreased in 38.4% of patients. Furthermore, 62.5% of patients stopped treatment due to a lack of clinical response or side effects experienced, 49% of whom withdrew within 12 weeks of starting treatment secondary to side effects. Key side effects resulting in cessation of treatment include hepatic dysfunction, interstitial lung disease, nausea, vomiting, decreased appetite, pneumonia, taste problems, diarrhea, neutropenia, and progression of disease during the first 12 weeks.

Some of the critical side effects (greater than Grade 3) were interstitial lung disease in 4.1% of patients and hepatotoxicity in 13.9%. Physicians should be aware of this and closely monitor patients from an early stage in treatment for these complications. Similarly, antiemetic drugs would be useful for patients to battle nausea and vomiting, a common side effect experienced.

Study limitations

There are several limitations of this systematic review. There were no strict selection criteria in terms of median age, gender, smoking status, type of NSCLC, time from diagnosis that crizotinib was started, presence of brain metastasis, and the line of treatment that the patient was on, all of which may affect treatment toxicities experienced. Other comorbidities that patients may have such as heart disease, diabetes, and other chronic conditions can significantly affect their ability to combat adverse effects and lower their threshold to withdraw from treatment, although a performance scale was used to pre-assess patients. The length of crizotinib treatment between each study was also not standardized, and patients could have experienced more side effects if the length of treatment was longer. Additionally, patients across each study would have had a different standardized performance score which could have affected their susceptibility to adverse effects. Furthermore, the cohort studies did not have a control group to compare treatment toxicity, which the three randomized clinical trials did.

This review excluded cross-sectional studies, meta-analyses, case reports and series, letters to the editor, and systematic reviews. These studies, if included, could have shed light on other adverse effects of crizotinib treatment that were not covered in this review.

Study strengths

This review summarized adverse effects across three randomized clinical trials and six cohort studies from various countries across the globe in the last 10 years. This allows us to understand the tolerability of crizotinib treatment in different cohort groups and gene pools, providing a more holistic view of the side effect profile of crizotinib. Some of these studies were the first crizotinib study to be conducted in the country, where data on crizotinib efficacy and safety profile was previously limited.

## Conclusions

Crizotinib is now one of several TKIs that have been approved by the FDA to treat ALK-mutated NSCLC. The nine studies reviewed show a lower percentage of adverse side effects and longer progression-free survival in the crizotinib group compared to patients treated with chemotherapy. However, alectinib showed lower toxicity and a better side effect profile than crizotinib, with a lower number of patients needing dose reductions or discontinuing treatment. Crizotinib only resulted in 9% of patients permanently withdrawing from treatment compared to 12% of patients treated with brigatinib.

For patients who experience adverse side effects when treated with 250 mg of crizotinib twice a day, dose reduction or temporary cessation are ways to tackle this while continuing treatment. This has been effective in patient groups experiencing QTc prolongation, bradycardia, or transaminitis to name a few. Clinicians should be aware of the adverse side effects of crizotinib, and how to combat these. Guidelines on how to titrate dosage based on side effects are available via the FDA.

Close monitoring of patients while on treatment and dose reduction or temporary cessation should be considered to tackle treatment toxicity and prevent early withdrawal from treatment. Despite these efforts, if patients are struggling with crizotinib treatment, other TKIs with a better side effect profile such as alectinib should be considered.
